# Periostin loss-of-function protects mice from post-traumatic and age-related osteoarthritis

**DOI:** 10.1186/s13075-021-02477-z

**Published:** 2021-04-08

**Authors:** Mukundan Attur, Xin Duan, Lei Cai, Tianzhen Han, Weili Zhang, Eric D. Tycksen, Jonathan Samuels, Robert H. Brophy, Steven B. Abramson, Muhammad Farooq Rai

**Affiliations:** 1 Division of Rheumatology, Department of Medicine, New York University Grossman School of Medicine, Langone Orthopedic Hospital, 550 1st Avenue, New York, NY 10016 USA; 2 Department of Orthopaedic Surgery, Washington University School of Medicine at Barnes-Jewish Hospital MS 8233, 425 South Euclid Avenue, St. Louis, MO 63110 USA; 3 Present address: Bluestone Center for Clinical Research, NYU College of Dentistry, New York, NY 10010 USA; 4 Genome Technology Access Center, McDonell Genome Institute, Washington University School of Medicine, St. Louis, 63110 MO USA; 5 Department of Cell Biology & Physiology, Washington University School of Medicine, St. Louis, 63110 MO USA

**Keywords:** Periostin, Knee osteoarthritis, Trauma, Aging, MMP-13, RNA-seq

## Abstract

**Background:**

Elevated levels of periostin (Postn) in the cartilage and bone are associated with osteoarthritis (OA). However, it remains unknown whether Postn loss-of-function can delay or prevent the development of OA. In this study, we sought to better understand the role of Postn in OA development and assessed the functional impact of *Postn* deficiency on post-traumatic and age-related OA in mice.

**Methods:**

The effects of *Postn* deficiency were studied in two murine experimental OA models using *Postn*^−/−^ (*n* = 32) and littermate wild-type (*wt*) mice (*n* = 36). Post-traumatic OA was induced by destabilization of the medial meniscus (DMM) in 10-week-old mice (*n* = 20); age-related OA was analyzed in 24-month-old mice (*n* = 13). Cartilage degeneration was assessed histologically using the OARSI scoring system, and synovitis was evaluated by measuring the synovial lining cell layer and the cells density in the synovial stroma. Bone changes were measured by μCT analysis. Serum levels of Postn were determined by ELISA. Expression of Postn and collagenase-3 (MMP-13) was measured by immunostaining. RNA-seq was performed on chondrocytes isolated from 21-day old *Postn*^−/−^ (*n* = 3) and *wt* mice (*n* = 3) to discover genes and pathways altered by *Postn* knockout.

**Results:**

*Postn*^−/−^ mice exhibited significantly reduced cartilage degeneration and OARSI score relative to *wt* mice in post-traumatic OA after 8 weeks (maximum: 2.37 ± 0.74 vs. 4.00 ± 1.20, *P* = 0.011; summed: 9.31 ± 2.52 vs. 21.44 ± 6.01, *P* = 0.0002) and spontaneous OA (maximum: 1.93 ± 0.45 vs. 3.58 ± 1.16, *P* = 0.014; summed: 6.14 ± 1.57 vs. 11.50 ± 3.02, *P* = 0.003). Synovitis was significantly lower in *Postn*^−/−^ mice than *wt* only in the DMM model (1.88 ± 1.01 vs. 3.17 ± 0.63; *P* = 0.039). *Postn*^−/−^ mice also showed lower trabecular bone parameters such as BV/TV, vBMD, Tb.Th, and Tb.N and high Tb. Sp in both models. *Postn*^−/−^ mice had negligible levels of serum Postn compared with *wt*. Immunofluorescent studies of cartilage indicated that *Postn*^−/−^ mice expressed lower MMP-13 levels than *wt *mice. RNA-seq revealed that cell-cell-adhesion and cell-differentiation processes were enriched in *Postn*^*−/−*^ mice, while those related to cell-cycle and DNA-repair were enriched in *wt* mice.

**Conclusions:**

*Postn* deficiency protects against DMM-induced post-traumatic and age-related spontaneous OA. RNA-seq findings warrant further investigations to better understand the mechanistic role of Postn and its potential as a therapeutic target in OA.

**Supplementary Information:**

The online version contains supplementary material available at 10.1186/s13075-021-02477-z.

## Introduction

Osteoarthritis (OA) is a clinical syndrome that affects more than 50 million people in the USA, with $185 billion in annual socioeconomic costs [1, 2]. Currently, there are no proven treatments to delay, let alone prevent, the progression of OA. Thus, a treatment that slows or halts disease progression before end-stage joint failure and arthroplasty is sorely needed. OA is recognized as a whole joint disease that affects all tissues (bone, cartilage, synovium, meniscus, and ligaments) [3, 4], although cartilage degeneration is considered the hallmark of end-stage disease [5]. Despite advances in preclinical studies, the pathways controlling early cartilage degeneration and new bone formation in OA remain unknown. Therefore, a critical issue in OA treatment is discovering early OA mediators, particularly elucidating the underlying intracellular signal transduction pathways, which affects multiple tissues in the joints.

Emerging evidence indicates periostin (Postn) is upregulated in patients with OA [6–9] and has been identified as a gene of interest in articular cartilage [10–12]. Postn is a vitamin K-dependent and glutamate-containing matricellular protein [13]. Postn, a member of the fasciclin family of proteins, was originally called osteoblast-specific factor 2 [14]. First identified in murine osteoblasts, it is also found in the periosteum [15]. Postn plays key roles in health and disease across various disciplines such as osteology and oncology [16–20]. It also plays a role in the repair mechanism of many connective tissues and organs such as the periodontal tissue, heart, and lungs [15, 16, 20–23]. Postn is considered important for its role in maintaining tissue integrity. In the bone, Postn is predominantly expressed in the periosteum and considered a key extracellular matrix (ECM) protein needed in health and healing. For instance, loss of Postn influences the propensity to fatigue fractures in mice [24], and Postn-deficient periosteum cannot reconstitute stem cells after injury [25]. Despite this evidence for the importance of Postn, it also has an adverse role in various inflammatory settings [26–28].

We have shown that expression of Postn is induced following joint trauma such as destabilization of the medial meniscus (DMM) or anterior cruciate ligament transection/partial medial meniscectomy and leads to development and progression of OA [12, 22]. Recently, our data confirmed that the Postn expression increases significantly in mouse and human cartilage and osteophytes during OA progression. Immune-localization studies further revealed that Postn was present in the cartilage ECM [10, 12, 22]. Emerging evidence supports the involvement of Postn in Wnt signaling activation and MMP-13 expression. Postn-induced MMP-13 expression was also inhibited by CCT031374 hydrobromide, an inhibitor of the canonical Wnt/β-catenin signaling pathway [12]. These findings suggest a catabolic role for Postn in promoting cartilage degeneration in OA by upregulating MMP-13 in response to injury.

In the present study, we hypothesize that genetic Postn loss-of-function in mice would protect against cartilage degeneration, synovitis, and bone changes secondary to aging and knee injury. To test this hypothesis, we assessed cartilage degeneration, synovial pathology, and bone morphometric parameters in two animal models. One consisting of 24-month-old mice with spontaneous OA and one with surgically induced OA model in 10-week-old mice. Postn knockout mice were compared to wild-type (*wt*) in both cohorts. We also performed RNA-seq on chondrocytes isolated from both *Postn*^−/−^ and *wt* mice in a pursuit to identify genes and pathways altered following *Postn* knockout.

## Methods

### Animals

In this study, we used homozygous mutant *Postn*^*tm1Jmol*^/J (*Postn*^*−/−*^*;* Stock No. 009067) mice on a B6129SF2/J (*wt;* Stock No. 101045) background. *Postn*^*−/−*^ and *wt* mice were obtained from The Jackson Laboratories (Bar Harbor, ME). Post-traumatic OA studies were performed at Washington University (St. Louis, MO), and aging studies were conducted at New York University Grossman School of Medicine (New York, NY). *Postn*^*−/−*^ mice were obtained by crossing heterozygous mice, and littermate mice from the same breeding were used for all experiments. All mice were kept and bred at the study institutions and genotyped by polymerase chain reaction (PCR) using tail deoxyribonucleic acid separated on 1.5% agarose gel using standard methods. Mice were housed in individually ventilated cages, with each cage having no more than five mice at a time in a hygienic barrier facility operating at 21–22 °C. Food and water were available *ad libitum*, and animals were maintained in a 12-h light, 12-h dark cycle. We used only male mice given sex differences in the DMM model [29]. The number of mice in each experimental group and each genotype are indicated in figure legends and summarized in Table 1.

**Table 1:** Distribution of mice for each experiment

Genotype	Age	Experiment	***n***
*Postn*^−/−^	10 weeks	DMM	10
	24 months	Aging	7
	24 months	Serum	6
	21 days	RNA-seq	3
	21 days	Real-time PCR	6
*wt*	10 weeks	DMM	10
	24 months	Aging	6
	24 months	Serum	11
	21 days	RNA-seq	3
	21 days	Real-time PCR	6

### Detection of Postn protein in the murine serum

Postn deficiency in *Postn*^−/−^ mice was detected by solid-phase enzyme-linked immunosorbent assay (ELISA). Briefly, mice euthanized by carbon dioxide asphyxiation were exsanguinated by cardiac puncture using mini-collect serum separator tubes (Greiner Bio-One, Monroe, NC). The blood was allowed to clot at room temperature for 30 min and retract at 4 °C for 4 h. The clotted blood was centrifuged at 2500 rpm at 4 °C for 10 min. The serum was collected, centrifuged, and stored at − 20 °C until used. Postn concentration in serum was measured using a mouse periostin/OSF-2 Quantikine ELISA kit according to the manufacturer’s instructions (MOSF20, R&D Systems Inc., Minneapolis, MN). In brief, 50 μl assay diluent was added to each well of a 96-well strip plate followed by an equal volume of standard, control, and sample in individual wells. The plate was incubated at room temperature on a horizontal orbital microplate shaker. After 2 h of incubation, the contents were removed, and each well was washed five times with the supplied wash buffer. Subsequently, 100 μl conjugate was added to each well followed by 2-h incubation at room temperature with constant shaking. Then, following washing as above, the plate was incubated with 100 μl substrate solution in each well for 30 min. Finally, the reaction was terminated with the addition of 100 μl of stop solution and the plate was read at 450 nm within 30 min.

### Induction of post-traumatic OA

To determine the role of Postn loss-of-function in post-traumatic OA, we performed the DMM surgery on 10-week-old male *Postn*^−/−^ or *wt* mice as described [30]. In short, mice were anesthetized with 2.5% isoflurane in 4 L/min oxygen. After aseptic preparation of the right hind limb, the joint capsule immediately medial to the patellar tendon was cut open, and the anterior attachment of the medial meniscotibial ligament was severed with sterilized microsurgical tools. This step resulted in the destabilization of the medial meniscus. Afterward, the joint capsule was closed with 6–0 absorbable polypropylene sutures (Ethicon, Blue Ash, OH), and the skin was closed by Vetclose skin glue (Henry Schein, Melville, NY). However, we used the contralateral left hind limb as non-operated control to avoid the effect of surgery. In compliance with guidelines, sustained-release buprenorphine (1.0 mg/kg) was administered once as an analgesic before surgery. No other pain-relieving medication was given to the mice. All mice were weight-bearing following recovery from the general anesthesia and resumed previous cage activity, water consumption, and food intake. Mice were euthanized 8 weeks after surgery by carbon dioxide inhalation. Hind limbs were separated, skinned, and subjected to histological and micro-computed tomography (μCT) analyses.

### Age associated spontaneous OA

For aging studies, 24-month-old *wt and Postn*^*−/−*^ mice were sacrificed using carbon dioxide asphyxiation; the hind limbs were separated, skinned, and prepared for histological and μCT analyses.

### Histological assessment of cartilage degeneration and OA

The knee joints were fixed in 10% neutral-buffered formalin for 48 h and decalcified using 12% formic acid, then embedded in paraffin using standard methods. Twelve coronal sections, each with 5 μm thickness, were taken from each joint at eight levels separated by 80 μm intervals. From each level, three sections were stained with Safranin O [31]. In each genotype, the same number and depth of sections were evaluated. The semi-quantitative Osteoarthritis Research Society International (OARSI) scoring system (scale: 0–6) was used to assess cartilage damage [32]. Cartilage damage was measured and scored in all four tibiofemoral compartments of the knee (lateral and medial femoral condyles and lateral and medial tibial plateaus) at all sectioned levels. The maximum OARSI score representing the highest score within all sectioned levels of the knee was recorded. Summed OARSI score was calculated by adding the total scores of four consecutive levels of each knee. Two independent scorers blinded to genotype and procedure scored the sections with a high inter-rater reliability.

### Histological assessment of synovitis

Safranin O-stained sections were graded for synovitis in the medial compartment for two parameters using a method developed by Lewis and colleagues [33]. Enlargement of the synovial lining cell layer is measured on a scale of 0–3, separately from the cells’ density in the synovial stroma on a scale of 0–3. Synovitis scores obtained for both of these parameters were averaged separately, and the sum of averages from both parameters used for analysis on a scale of 0–6.

### Immunostaining for Postn and MMP-13

Histological sections were deparaffinized using xylene and then rehydrated in a graded series of ethyl alcohol. Endogenous peroxidase activity quenched by incubating slides with 3% (v/v) hydrogen peroxide in phosphate-buffered saline (PBS). After 15 min of incubation, proteinase K (10 μg/mL, Abcam, Cambridge, MA) was added to the sections for 20 min at 37 °C to retrieve the antigen. Following washing with PBS and blocking with 10% normal goat serum (NGS), slides were allowed to react overnight at 4 °C with the following primary antibodies diluted in 2% NGS: anti-periostin (1:100, Sigma-Aldrich, St. Louis, MO), MMP-13 (1:200, Abcam), and in-house collagen type II (Col 2, 1:200). The next day, following the washing step, slides were incubated with the corresponding HRP- (for Postn), Alexa 488-(MMP-13), or Alexa 594-(for Col 2) conjugated secondary antibody in 2% NGS for one-hour room temperature. Then, slides (MMP-13 and Col 2) underwent counterstaining with Fluoro-Gel II with 4′,6-diamidino-2-phenylindole (DAPI, Electron Microscopy Sciences, Hatfield, PA) for MMP-13 and Col 2. For immunohistochemical staining of Postn, HRP stained slides were developed with 3,3’-Diaminobenzidine (DAB Chromogen Kit; Vector Laboratories Inc., Burlingame, CA) for 10 min at room temperature. All images were visualized using a NanoZoomer (Hamamatsu Corp., Bridgewater, NJ) or Confocal Laser Scanning Microscope (Leica, Biosystems, Buffalo Grove, IL). MMP-13 expression levels were quantified by measuring the staining intensity of 20–40 cells in each stained section using LAS X software (Leica Biosystems).

### μCT analysis of the trabecular bone

After fixation but before decalcification, the knees undergoing DMM were scanned using a vivaCT 40 in vivo μCT scanner (Scanco Medical Inc., Southeastern, PA) with the following setting: voxel size = 21 μm, energy = 45 kV, intensity = 177 μA, and integration time = 300 ms [34]. To analyze bone changes, the femoral epiphysis was chosen as the region of interest. The region of interest was identified between the cartilage and the growth plate. The outline of the epiphysis was carefully selected without the inclusion of outgrowing osteophyte(s). Knees from 24-month-old mice were scanned using a 10 MP digital detector using the following parameters: 10 W of energy (50 kV and 200 mA), pixel size = 9.7 μm, exposure = 1025 ms/frame, rotation step 0.3 degrees with × 10 frames averaging, 0.5 mm Aluminum filter, and scan rotation = 180^o^. After scanning, the radiographs were reconstructed using NRecon software ver. 1.7.3.0 (Bruker μCT, Kontich, Belgium). Reconstruction was done with NRecon using GPU acceleration. Gaussian smoothing was applied with a 2-voxel radius, ring artifact, and beam hardening corrections were applied in reconstruction. Ring artifact reduction set to 7 pixels. Beam hardening correction was set to 40%. Following trabecular bone morphometric parameters defined by the American Society for Bone and Mineral Research were analyzed [35]: bone volume fraction (BV/TV), volumetric bone mineral density (vBMD), trabecular thickness (Tb.Th), trabecular number (Tb.N), and trabecular spacing (Tb.Sp).

### RNA-Seq analysis, gene ontology annotation, and transcript expression validation

We performed bulk RNA-seq on articular chondrocytes isolated from *Postn*^*−/−*^ and *wt* mice to determine the baseline transcript-level differences between the two genotypes.

#### Chondrocyte isolation and culture

Primary chondrocytes were isolated from femoral head cartilage of 21-day-old *Postn*^*−/−*^ (*n* = 3) and *wt* (*n* = 3) mice as described previously [36]. The cells were seeded in 24-well plates, supplied with 10% fetal bovine serum (FBS; Thermo Fisher Scientific, Waltham, MA) in high glucose Dulbecco’s modified Eagle’s medium (DMEM; Thermo Fisher Scientific) supplemented with 1% penicillin and streptomycin (10,000 U/mL and 10,000 ug/mL respectively;, Thermo Fisher Scientific) and incubated at 37 °C in a humidified incubator with 5% CO_2_. After 2 days of culture, cells were washed 3× with PBS before RNA extraction.

#### RNA extraction

Total RNA was extracted from 1.0 × 10^5^ cells using a column-based RNeasy Mini kit (Qiagen, Valencia, CA). Total RNA quality and concentrations were measured using Agilent Bioanalyzer (Agilent Technologies Inc., Santa Clara, CA). RNA samples with a RIN (RNA integrity number) score > 9.0 were used for RNA-seq analysis and real-time PCR.

#### Library preparation, sequencing, and gene ontology analysis

Library preparation was performed with 10 ng of total RNA. Double-stranded cDNA was prepared using Clontech SMARTer Ultra Low RNA kit (Takara Bio Inc., Mountain View, CA). cDNA was fragmented with an E220 sonicator (Covaris Inc., Woburn, MA) using these settings: peak incident power = 18, duty factor = 20%, and cycles per burst = 50 for 120 s. cDNA was blunt-ended, had an A base added to the 3′ ends, and had Illumina sequencing adapters ligated to the ends. Ligated fragments were amplified for 12–15 cycles using primers incorporating unique dual index tags. Fragments were sequenced on an Illumina HiSeq 3000 (Illumina, San Diego, CA) using single-end reads extending 50 bases. Base calls and demultiplexing performed with Illumina’s bcl2fastq software, and a custom python demultiplexing program with a maximum of one mismatch in the indexing read. RNA-seq reads were aligned to the Ensembl release 76 top-level assemblies with STAR version 2.0.4b [37]. Gene counts were derived from the number of uniquely aligned unambiguous reads by Subread:featureCount version 1.4.5 [38]. Sequencing performance was assessed for the total number of aligned reads, the total number of uniquely aligned reads, and features detected. The ribosomal fraction, known junction saturation, and read distribution over known gene models were quantified with RSeQC version 2.3 [39].

All gene counts imported into the R/Bioconductor package EdgeR [40], and TMM normalization size factors were calculated to adjust for samples for differences in library size. Ribosomal genes and genes not expressed in the smallest group size minus one sample greater than one count-per-million excluded from further analysis. The TMM size factors and the matrix of counts were then imported into the R/Bioconductor package Limma [41]. Weighted likelihoods based on the observed mean-variance relationship of every gene and sample were then calculated for all samples with the voomWithQualityWeights [42] function with additional unknown latent effects as determined by surrogate variable analysis [43]. All genes performance assessed with plots of the residual standard deviation of every gene to their average log-count with a robustly fitted trend line of the residuals. Differential expression analysis was performed to analyze for differences between conditions, and the results were filtered for only those genes with Benjamini-Hochberg false-discovery rate (FDR) adjusted *P* ≤ 0.05.

For each contrast extracted with Limma, global perturbations in known Gene Ontology (GO) terms detected using the R/Bioconductor package “Generally Applicable Gene set Enrichment” [44] to test for changes in expression of the reported log_2_-fold changes reported by Limma in each term versus the background log_2_-fold of all genes found outside the respective term. The R/Bioconductor package heatmap3 [45] used to display heatmaps across groups of samples for each GO term with a Benjamini-Hochberg FDR-adjusted *P* ≤ 0.05.

#### Validation of RNA-seq data by quantitative real-time PCR

We validated RNA-seq data by quantitative real-time PCR. We selected representative genes for each pattern of expression. Expression of *Dscaml1* and *Tm4sf1* was higher in *Postn*^*−/−*^ and *wt* mice respectively, expression of *Ndufs5* and *Srsf10* was similar in both groups. Total RNA was prepared from chondrocytes isolated from *Postn*^*−/−*^ mice *(n* = 6) and *wt* mice (*n* = 6) mice. A total of 250 ng of total RNA was subjected to Amplification Grade DNase I treatment (1 U/μL, Thermo Fisher Scientific), to eliminate traces of genomic DNA and then reverse transcribed to synthesize the first strand of cDNA (High-Capacity Reverse Transcription Kit, Thermo Fisher Scientific). Briefly, 2 μL cDNA was added to the reaction mixture comprising of 2× SYBR Green Master Mix (Thermo Fisher Scientific), gene-specific forward and reverse primers (primer sequences are depicted in Table 2), and RNase-free water to reach a total volume of 20 μL. Subsequently, real-time PCR was performed under the following settings: 94 °C for 3 min, followed by 40 cycles at 94 °C for 30 s, 60 °C for 30 s, and 72 °C for 30 s. The target gene expression was normalized to the housekeeping gene, *Gapdh*. Each analysis was performed in triplicate. The relative expression values were computed using 2^−ΔΔ^Ct method.

**Table 2:** Sequence and characteristics of primers used for real-time PCR

Gene symbol	Forward primer (5′ → 3′)	Location	Reverse primer (5′ → 3′)	Location	Amplicon size	Accession no.
*Dscaml1*	aggctgaagaggctacgaga	4972–4991	gaggtcctttcacaggggtg	5062–5043	91 bp	NM_001081270.2
*Tm4sf1*	actgggtttggcagaaggac	1006–1025	tgggctcatagcacttggac	1127–1108	122 bp	NM_008536.4
*Ndufs5*	acagccctataagaacgccg	148–167	tgtaccgaagcaagcactct	287–268	140 bp	NM_001030274.1
*Srsf10*	acgtcgggaatttggtcgtt	234–253	agcgtcttcagcatcacgaa	357–338	124 bp	NM_010178.3
*Gapdh*	aggtcggtgtgaacggatttg	100–120	tgtagaccatgtagttgaggtca	222–200	123 bp	NM_001289726.1

*bp* = base pair

### Statistical analysis

The non-parametric Mann-Whitney test used to compare the data from two genotypes (*wt* and *Postn*^−/−^) unless indicated otherwise. Results were considered statistically significant at *P* < 0.05. Data are presented as mean ± standard deviation with 95% confidence interval (CI) where indicated. All statistical analyses were performed using GraphPad PRISM version 7.03 (GraphPad Software Inc., San Diego, CA).

## Results

### *Postn*^*−/−*^ mice exhibit no Postn in the joint and in the serum

**Fig. 1:**
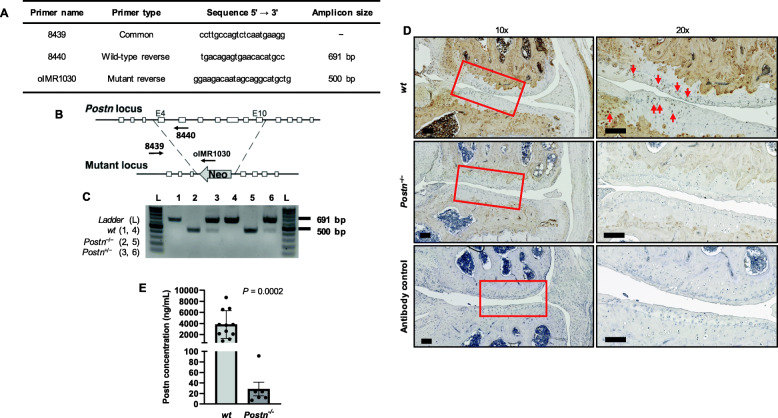
*Postn*^−/−^ mice lack Postn protein. **a** Primers used for mouse genotyping. **b** Diagram of targetting vector for genertaion of *Postn*^−/−^ mice. **c** Genotyping analysis of *wt*, *Postn*^−/−^ and *Postn*^+/−^ mice. **d** Immunostaining of Postn in the knee joint revealed that *wt* (*n* = 3) mice expressed periostin (red arrows) whereas *Postn*^−/−^ (*n* = 3) mice showed no staining for Postn (left panel: 10x, right panel 20x). Scale bar = 100 μm. **e** Serum Postn levels were negligible and significantly low in *Postn*^−/−^ (*n* = 6) than *wt* mice (*n* = 11) (Mann-Whitney test)

The genotype of each mouse was confirmed by PCR (Fig. 1a–c). We demonstrated a complete loss of Postn protein in *Postn*^−/−^ mice by immunostaining of the joint section (Fig. 1d) and ELISA in serum. ELISA results revealed significantly low levels of Postn protein in the serum of *Postn*^*−/−*^ mice compared with the *wt* mice (28.33 ± 21.65 [95% CI = − 4.88–61.55] vs. 3827.00 ± 2504.00 [95% CI = 2144.00–5509.00], *P* = 0.0002) (Fig. 1e).

### *Postn*^*−/−*^ mice develop less severe OA after DMM

#### Cartilage degeneration

**Fig. 2:**
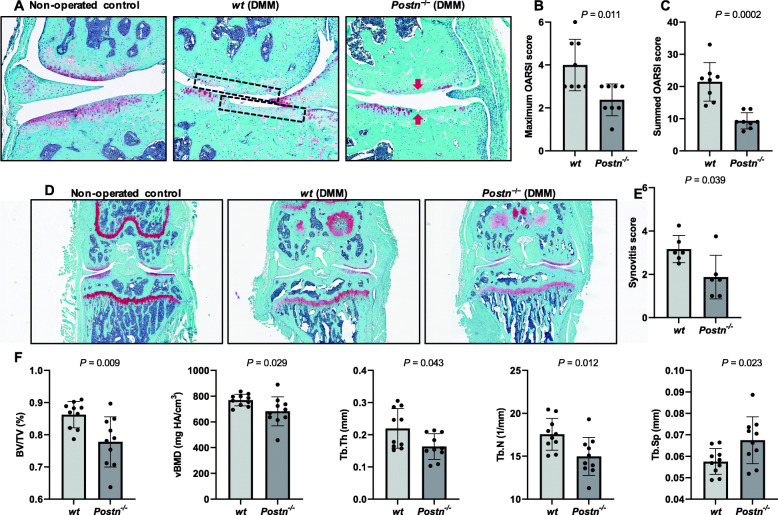
*Postn*^−/−^ mice are protected from post-traumatic OA. **a** Histological analysis of the cartilage showed that DMM-operated limbs exhibited increased cartilage degeneration than non-operated control limbs. Cartilage degeneration in *wt* mice was markedly higher (dotted boxes) than *Postn*^−/−^ mice (arrows). **b** Semi-quantitative analysis of cartilage degeneration by maximum OARSI score showed that *Postn*^−/−^ mice (*n* = 8) had significantly less maximum OARSI score than *wt* mice (*n* = 8). (Mann-Whitney test). **c** Semi-quantitative analysis of cartilage degeneration by summed OARSI score showed that *Postn*^−/−^ mice (*n* = 8) had significantly less summed OARSI score than *wt* mice (*n* = 8). (Mann-Whitney test). **d** Synovitis was evaluated by measuring synovial lining cell layer and density of cells in the synovial stroma. **e** Semi-quantification of synovitis score was significantly lower in *Postn*^−/−^ mice (*n* = 6) than *wt* mice (*n* = 6). (Mann-Whitney test). **f** μCT analysis showed that *Postn*^−/−^ mice (*n* = 10) had significantly lower BV/TV. vBMD, Tb.Th and Tb.N and significantly higher Tb. Sp than *wt* mice (*n* = 10) in the DMM-operated limb (Mann-Whitney test)

The analysis of histological sections of DMM-operated or non-operated knees from *Postn*^*−/−*^ and *wt* mice at 8-weeks after DMM surgery (Fig. 2a) showed that non-operated control mice retained relatively normal cartilage with maximum OARSI score ≤ 1.00. Conversely, many histological features representative of OA were apparent 8 weeks after surgery in *wt* mice: reduced Safranin O staining depicting proteoglycan loss in the ECM, fibrillation, and delamination of superficial zone cartilage, and in severe cases, an extension of the cleft lesions into the middle zone. These features illustrate that DMM surgery-induced degeneration of the cartilage resembles human OA pathology. The mean maximum OARSI score for *wt* mice in DMM-operated knees (4.00 ± 1.20; 95% CI = 3.00–5.00) was significantly (*P* < 0.001) higher than that of non-operated control knees (0.75 ± 0.46; 95% CI = 0.36–1.14). *Postn*^−/−^ mice that underwent DMM showed significantly less cartilage damage with the mean maximum OARSI score of 2.37 ± 0.74 (95% CI = 1.75–3.00), significantly (*P* = 0.011) lower than for their littermate *wt* mice (4.00 ± 1.20; 95% CI = 3.00–5.00) (Fig. 2b). Likewise, DMM-operated *Postn*^−/−^ mice (9.31 ± 2.52, 95% CI = 7.21–11.42) had significantly (*P* = 0.0002) lower mean summed OARSI score than *wt* mice (21.44 ± 6.01, 95% CI = 16.41–26.46) (Fig. 2c).

#### Synovitis

We noted a thinner synovial lining cell layer and a low density of cells in the synovial stroma in *Postn*^−/−^ mice than *wt* mice (Fig. 2d). Quantification of synovitis score was significantly (*P* = 0.039) lower in *Postn*^−/−^ mice (1.88 ± 1.01; 95% CI = 0.82–2.93) than *wt* mice (3.17 ± 0.63; 95% CI = 2.51–3.82) (Fig. 2e).

#### Trabecular bone changes

Interestingly, there were no differences in the bone parameters measured in the control/contralateral limbs between the genotypes (Table 3). The differences in the various parameters of trabecular bone between *wt* and *Postn*^*−/−*^ are shown in Fig. 2f. We noted that the following bone parameters were significantly lower in *Postn*^*−/−*^ mice compared with *wt* mice: trabecular BV/TV (9.8%, *P* = 0.009), vBMD (11.3%, *P* = 0.029), Tb.Th (25.6%, *P* = 0.043), and Tb.N (14.6%, *P* = 0.012). In contrast, Tb. Sp was significantly higher in *Postn*^*−/−*^ mice than *wt* mice (17.2%, *P* = 0.023).

**Table 3:** Bone epiphysial parameters in control limbs of wt and *Postn*^−/−^mice

Parameter	***wt***		***Postn***^**−/−**^		***P value***
	Mean ± SD	95% CI	Mean ± SD	95% CI	
BV/TV (mm^3^/mm^3^)	0.79 ± 0.04	0.76–0.82	0.73 ± 0.09	0.66–0.79	0.143
vBMD (mg HA/cm^3^)	650.70 ± 57.35	609.70–691.70	608.90 ± 103.60	534.70–682.90	0.436
Tb.Th (mm)	0.20 ± 0.11	0.13–0.28	0.15 ± 0.03	0.13–0.17	0.248
Tb.N (1/mm)	14.46 ± 2.54	12.64–16.28	15.33 ± 2.87	13.28–17.39	0.631
Tb.Sp (mm)	0.07 ± 0.02	0.06–0.08	0.06 ± 0.01	0.05–0.07	0.353

*SD* standard deviation, *CI* confidence interval

### 24-month-old *Postn*^*−/−*^ mice demonstrated protection from spontaneous OA

#### Cartilage degeneration

**Fig. 3:**
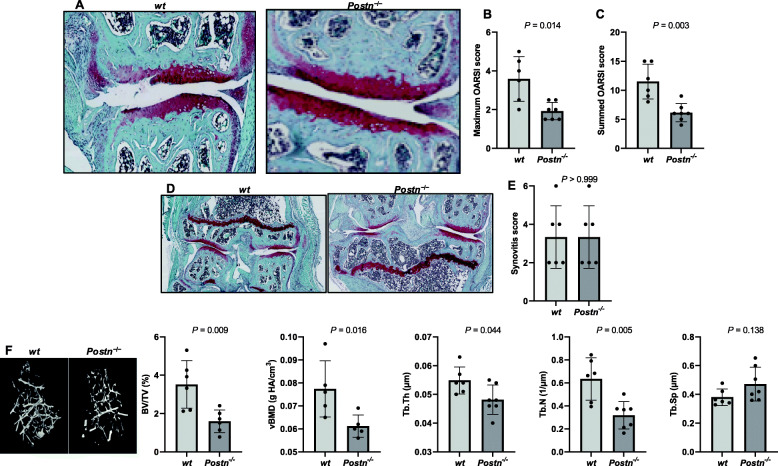
*Postn*^−/−^ mice are protected from age-related spontaneous OA. **a** Histological analysis of cartilage showed that *wt* mice developed significant cartilage degeneration at 24-month of age whereas age-matched *Postn*^−/−^ mice did not develop any degeneration in the articular cartilage. **b** Semi-quantitative analysis of cartilage degeneration using maximum OARSI score displayed that *Postn*^−/−^ mice (*n* = 7) had significantly less maximum OARSI score than *wt* mice (*n* = 6) (Mann-Whitney test). **c** Semi-quantitative analysis of cartilage degeneration using summed OARSI score displayed that *Postn*^−/−^ mice (*n* = 7) had significantly less summed OARSI score than *wt* mice (*n* = 6) (Mann-Whitney test). **d** Synovitis was assessed by semi-quantiative analysis of synovial lining cell layer and density of cells in the synovial stroma. **e** Semi-quantification of synovitis score was not significantly different between the genotypes (*n* = 6 each). (Mann-Whitney test). **f** μCT analysis showed that *Postn*^−/−^ mice (*n* = 5–7) had significantly lower BV/TV, vBMD, Tb.Th, and Tb.N and higher Tb. Sp than *wt* mice (*n* = 5–6) in the DMM-operated limbs (Mann-Whitney test)

Representative histological sections from 24-month-old *Postn*^*−/−*^ and *wt* mice are shown in Fig. 3a. Histological analysis of the knee joints of 24-month-old *Postn*^*−/−*^ and *wt* mice showed that *Postn*^−/−^ mice had less cartilage degeneration (only focal loss of proteoglycans without cartilage loss) than *wt* mice, which exhibited increased loss of cartilage proteoglycan and delamination of both superficial and middle zones. These observations confirmed that in mice, aging induces spontaneous cartilage degeneration that resembles OA pathology in humans. The mean maximum OARSI score in *Postn*^*−/−*^ mice was significantly lower than in *wt* mice (1.93 ± 0.45 [95% CI = 1.51–2.35] vs. 3.58 ± 1.16, [95% CI = 2.37–4.80], *P* = 0.014) (Fig. 3b). Similarly, the mean summed OARSI score was significant (*P* = 0.003) lower in *Postn*^*−/−*^ mice (6.14 ± 1.57, 95% CI = 4.69–7.60) than *wt* mice (11.50 ± 3.02, 95% CI = 8.33–14.67) (Fig. 3c).

#### Synovitis

The synovial lining cell layer and cell density in the synovial stroma appeared similar in both genotypes (Fig. 3d). We did not find any significant (*P > *0.999) difference in synovitis score between *Postn*^−/−^ mice (3.33 ± 1.63; 95% CI = 1.62–5.05) and *wt* mice (3.33 ± 1.63; 95% CI = 1.62–5.05) **(**Fig. 3e).

#### Trabecular bone changes

The changes in different trabecular bone parameters are depicted in Fig. 3f. We observed that *Postn*^*−/−*^ mice exhibited significantly lower trabecular BV/TV (54.7%, *P* = 0.009), vBMD (20.9%, *P* = 0.016), Tb.Th (12.2%, *P* = 0.044) and Tb.N (49.7%, *P* = 0.005) in contrast to *wt* mice. Also, Tb. Sp was 24.0% higher in *Postn*^*−/−*^ than *wt* mice but did not reach significance (*P* = 0.138).

### MMP-13 expression decreased in *Postn*^*−/−*^ mice

**Fig. 4:**
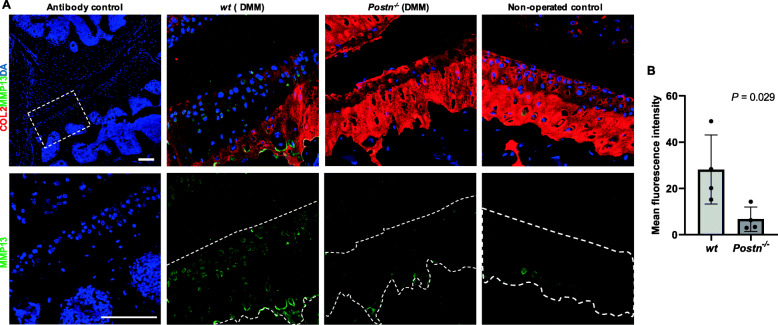
*Postn*^−/−^ mice expressed less MMP-13 than *wt* mice after DMM. **a** Immunofluorescent analysis showed that MMP-13 staining (green) was higher in cartilage of DMM-operated limbs than in the non-operated control limb. *Postn*^−/−^ mice (*n* = 4) showed less staining of MMP-13 than *wt* mice (*n* = 4). Type II collagen (Col 2) (red) staining was used for location of cartilage. The dotted lines on lower panels indicate the Col 2-positive cartilage area based on the results in the upper panels. DAPI (blue) was used as a counterstain. Scale bars = 100 μm. **b** Quantification of immunostaining intensity of MMP-13 showed that MMP-13 intensity was significantly lower in *Postn*^−/−^ mice (*n* = 4) than *wt* mice (*n* = 4). (Mann-Whitney test)

Immuno-fluorescence analysis of MMP-13 revealed that its expression was increased in *wt* mice following DMM compared with the control knees. In contrast, no MMP-13 was detected in DMM-operated knees of *Postn*^*−/−*^ mice (Fig. 4a). Semi-quantitative assessment of immunofluorescence imaging further showed that intensity of MMP-13 staining was significantly (*P* = 0.029) lower in *Postn*^*−/−*^ (6.73 ± 5.23, 95% CI = -1.60–15.06) than *wt* mice (28.18 ± 14.94, 95% CI = 4.42–51.95) (Fig. 4b).

### RNA-seq and gene ontology analyses

**Fig. 5:**
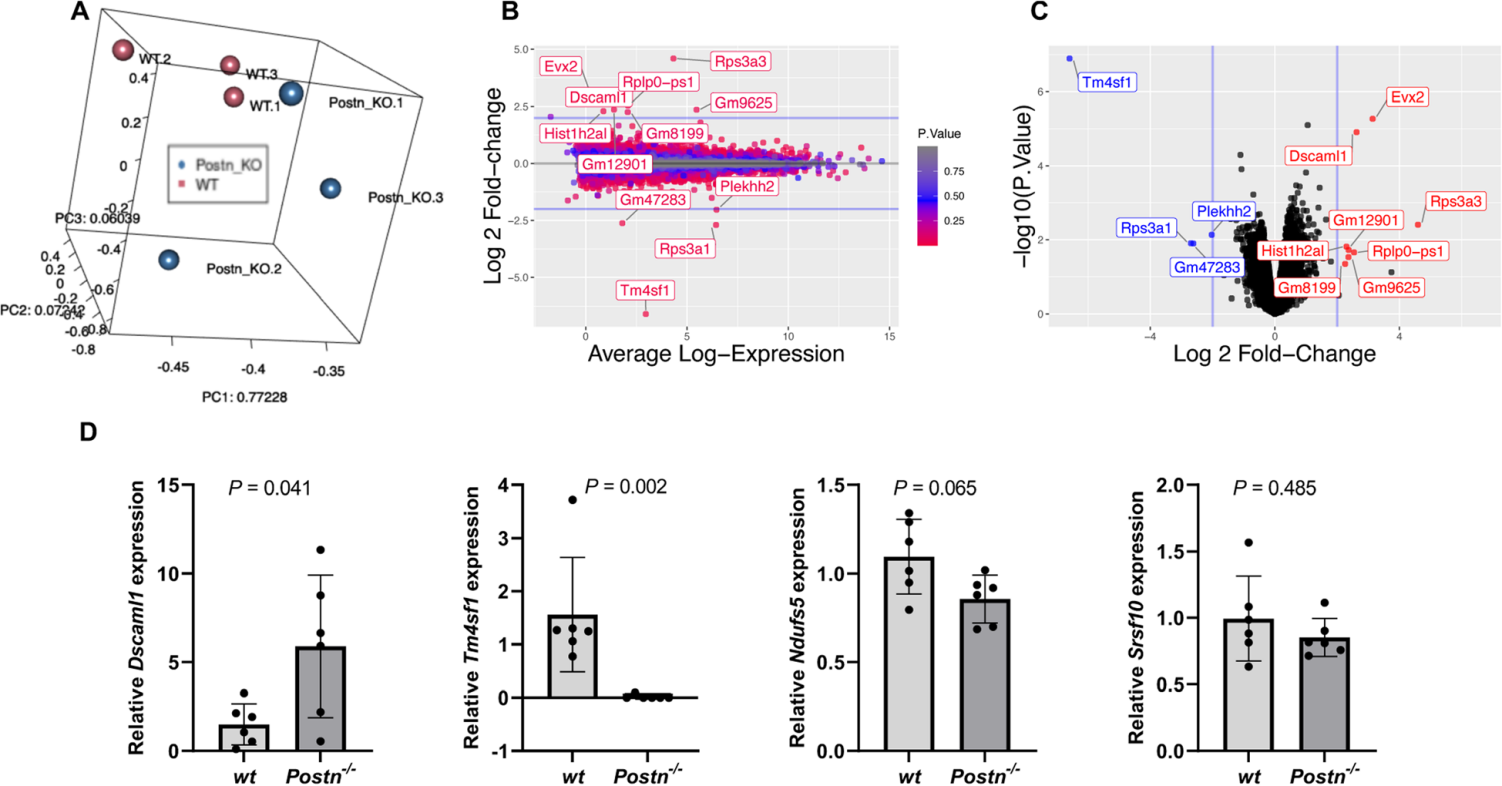
Descriptive RNA-seq data and real-time PCR. **a** Principal component analysis of chondrocytes samples from *wt* (*n* = 3) and *Postn*^−/−^ *(n* = 3) mice showed distinct grouping based on gene expression differences. **b** MA (log_2_ ratio to mean expression) plot depicts expression fold change and averaged expression level of differentially expressed transcripts. **c** Volcano plot of all genes expressed greater than 1 count-per-million in all 6 samples where the observed log_2_ fold change is on the *x*-axis and the unadjusted *P* value converted to the −log_10_ scale is on the *y*-axis. Twelve genes with unadjusted *P* ≤ 0.05 are highlighted in red when log_2_ fold changes > + 2 and blue when < – 2. **d** Real-time PCR performed on chondrocytes isolated from an independent cohort of *wt* (*n* = 6) and *Postn*^−/−^ (*n* = 6) mice revealed an agreement with the RNA-seq data. (Mann Whitney test)

Qualitative gene expression analysis revealed that samples were clustered into two distinct clusters of *Postn*^*−/−*^ and *wt* chondrocytes based on principal component analysis indicating a specific expression profile (Fig. 5a) though there was higher variation among samples from *wt* mice than *Postn*^*−/−*^ mice. Moreover, we show that expression fold change and average expression levels vary subtly in a mean average (log_2_ ratio to mean expression plot) (Fig. 5b).

#### Detection of differentially expressed gene transcripts

Gene transcripts (Supplemental Table 1) were significantly differentially expressed (*n* = 1247) between *Postn*^*−/−*^ and *wt* chondrocytes at an unadjusted *P* ≤ 0.05 with 727 being upregulated in *Postn*^*−/−*^ while 520 were upregulated in *wt* chondrocytes. Out of 1247, only 48 transcripts had log_2_ fold-change ≥2. After, FDR ≤ 0.05 correction, only four transcripts (Table 4) were found to be differentially expressed (3 upregulated and 1 downregulated) between *Postn*^*−/−*^ compared with *wt*. Volcano plot of genes expressed greater than 1 count-per-million in all six samples is shown (Fig. 5c).

**Table 4:** Gene transcripts differentially expressed in chondrocytes between *Postn*^−/−^ and *wt* mice

Gene symbol	Gene name	Log_**2**_ fold change	***P***	Description
*Evx2*	Even skipped homeobox 2	3.14	5.30 × 10^− 6^	Up-regulated in *Postn*^−/−^ mice
*Dscaml1*	DS cell adhesion molecule like 1	2.62	1.22 × 10^− 5^	Up-regulated in *Postn*^−/−^ mice
*Gdf10*	Growth differentiation factor 10	1.05	7.85 × 10^−6^	Up-regulated in *Postn*^−/−^ mice
*Tm4sf1*	Transmembrane 4 superfamily member 1	−6.59	1.27 × 10^− 7^	Down-regulated in *Postn*^−/−^ mice

#### Gene ontology annotation

Gene ontology analysis revealed that a number of distinct biological processes were significantly enriched for each genotype (Table 5). While the biological processes related to cell-cell adhesion, cell signaling, cell differentiation, focal adhesion, and angiogenesis were elevated in *Postn*^*−/−*^ mice, biological processes such as cell cycle, cell division, and DNA repair repressed in *Postn*^*−/−*^ mice.

**Table 5:**
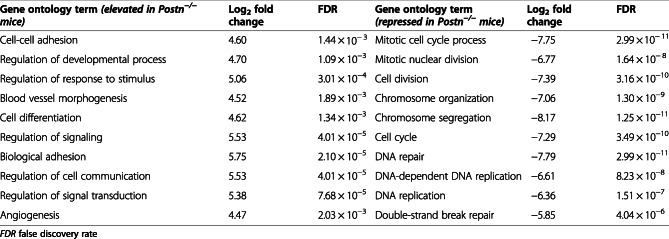
Biological processes (gene ontologies) altered in chondrocytes between *Postn*^−/−^ and *wt* mice

#### Validation by real-time PCR

We further confirmed the differentially expressed genes by quantitative real-time PCR in agreement with the RNA-seq data (Fig. 5d).

## Discussion

Our study illustrates that *Postn* deficiency exerts a protective effect against OA in mice. Specifically, mice lacking the *Postn* gene displayed significantly less cartilage degeneration than *wt* control mice in post-traumatic OA as well as age-related primary OA. Postn deficiency also appears to wield protective effects by modulating changes in the synovium and bone. Thus, our findings highlight that Postn loss-of-function protects against incidence and progression of OA independent of the cause, i.e., primary age-related spontaneous OA as well as injury-induced post-traumatic OA.

In both mouse models of OA, *Postn*^*−/−*^ mice retained superior cartilage and exhibited less degenerative changes as measured by OARSI scores. These findings are consistent with the notion that protection from cartilage degeneration parallels protection from OA since cartilage degeneration is considered OA’s hallmark [4]. Besides, we also showed that *Postn*^*−/−*^ mice developed significantly less synovitis than *wt* mice after DMM. Tajia et al. have also shown increased expression of Postn in human OA synovium [46]. These protective effects of *Postn* loss-of-function on the cartilage and synovium indicate decreased catabolic activity in the joint. Our observation aligns with our previous in vitro work showing that siRNA-mediated knockdown of Postn in chondrocytes results in reduced expression of inflammatory and catabolic markers such as IL-1β, ADAMTS-4/5, and MMP-13 [12, 22].

We also found differences in trabecular bone parameters between the genotypes (*Postn*^−/−^ and *wt*) for both OA models. BV/TV, vBMD, Tb.Th, and Tb.N was low and Tb. Sp was high in *Postn*^*−/−*^ mice. These parameters are not only standard osteoporosis measurements but have also been used as markers for OA [47–49]. The pattern of these bony changes in *Postn*^*−/−*^ mice mirrors OA protection as has been noted in rodent and human studies of OA [31, 47–52]. These findings suggest that Postn plays a role in modulating changes in cartilage as well as in the bone. However, it is not possible to conclude that the bone is driving the cartilage phenotype from this investigation.

In the present study, we did not examine how the genetic deficiency of *Postn* protects mice from developing OA. However, several studies suggest a possible link connecting the mechanisms of Postn-mediated cartilage degeneration with NF-κB and Wnt signaling [7, 12, 22, 53]. We have previously shown that Postn expression is increased following injury [12]. Postn is upregulated in human OA cartilage and cartilage from mice undergoing DMM and is correlated with increased MMP-13 expression in OA cartilage [12, 22]. Increased expression of Postn leads to overexpression of cartilage ECM degrading enzymes such as MMP-13 and ADAMTS-5. Postn also promotes condylar resorption via the NF-κB-ADAMTS-5 axis in temporomandibular joint OA [54]. Overexpression of Postn by the administration of recombinant proteins or through lentivirus transduction leads to increased expression of MMP-13 in human and murine chondrocytes. Likewise, Postn inhibition or deficiency results in decreased expression of catabolic enzymes. Therefore, we surmise that loss of *Postn* protects against cartilage degeneration by decreasing ECM degrading enzymes such as MMP-13 and ADAMTS-5. A recent study identified DDR-1 as a potential receptor for Postn-mediated signaling in chondrocytes. It showed that blocking DDR-1 with small chemical inhibitors reduced MMP-13 expression and cartilage degradation in vitro and in vivo [53].

Baseline transcriptomic differences between *Postn*^*−/−*^ and *wt* mice revealed interesting findings. The expression of *Evx2*, *Dscaml1*, and *Gdf10* increased in *Postn*^*−/−*^ compared with *wt* mice. *Evx2* is predominantly expressed in the limbs and is involved in vertebrate limbs’ morphogenesis, where it interacts with Hox genes [55]. *Dscaml1* is a member of the immunoglobulin superfamily of cell adhesion molecules, which participates in neuronal differentiation [56]. Tuure et al. reported that the expression of *Dscaml1* is low in human OA chondrocytes treated with IL-1β and is increased following treatment with a selective inhibitor of microsomal prostaglandin E synthase 1 [57]. *Gdf10* gene encodes a secreted ligand of transforming growth factor-beta superfamily of proteins, which plays essential functions in chondrocyte differentiation and bone formation [58]. In particular, it was found that the expression of *Gdf10* was increased in chondrocytes under hypoxia, where *Gdf10* was regulated by Sox-9, suggesting its protective role in chondrocytes [59]. The expression of *Tm4sf1* was decreased in *Postn*^*−/−*^ mice*. Tm4sf1* is a surface marker, known to inhibit apoptosis and promote cell proliferation and migration [60]. This transcriptomic profile paralleled with gene ontology annotations. For instance, the biological processes related to cell-cell adhesion, cell signaling, cell differentiation, and focal adhesion were elevated in *Postn*^*−/−*^ mice, while biological processes such as cell cycle, cell division, and proliferation were repressed in *Postn*^*−/−*^ mice. Together, these findings provide novel insights into the role of Postn in chondrocytes, specifically highlighting that increased expression of *Dscaml1* and *Gdf10* (and related biological processes) offers protection from OA, while the expression of *Tm4sf1* (and related biological processes) related to cellular phenotypes that are altered with Postn knockout. While the role of Postn in OA has been discussed in the previous paragraph, here, we highlight its role in cell functions. Chinzei et al. showed that exogenous overexpression of Postn increases chondrocyte migration which is impeded by its knockdown [22]. Likewise, Padial-Molina et al. reported an increase in proliferation and migration of periodontal ligament fibroblasts with Postn treatment [61]. Finally, Cai and colleagues showed that Postn knockdown decreases cell matrix [23]; however, no data are yet available for cell-cell-adhesion in the context of Postn knockdown or overexpression in chondrocytes.

Our finding that *Postn* deficiency reduces both age-related spontaneous OA and post-traumatic OA is important. While aging and obesity are associated with primary idiopathic OA [62–65], joint injuries cause post-traumatic OA cases [66, 67] and constitute at least 12% of OA [68]. While both OA forms are qualitatively similar in that both share standard features such as cartilage degeneration and bone sclerosis [69], the underlying disease mechanisms are dissimilar in many ways [10]. However, their degree of overlap is unclear, particularly at the mechanistic level. Our finding that Postn deficiency protected the joint from cartilage degeneration and bone alterations in both types of OA suggests that Postn ablation may have widely applicable therapeutic efficacy. Specifically, anti-Postn therapy may have utility for primary idiopathic OA as well as for post-traumatic OA.

In this study, we have shown that global deletion of Postn is protective against OA. Postn knockout is embryonically non-lethal, yet some *Postn*^*−/−*^ mice die shortly after birth. Surviving mice have 10–20% growth retardation and exhibit some skeletal abnormalities such as shorter subchondral bone, weaker ligaments, and moderate scoliosis (Cai et al. unpublished data), which complicate mechanistic exploration in a specific tissue, cartilage, for example. Thus, in future studies, we will disable Postn in a temporal (inducible) and cartilage-specific manner by genetic ablation in mature mice to study OA development.

This study has some limitations, we did not report data from heterozygotes (*Postn*^*+/−*^) since no protective effect was observed in those mice. Other limitations included the lack of measurement of functional outcomes such as behavior, pain, and gait as well as the lack of assessment of progressive changes as the only one-time point was studied in both models. Moreover, detailed analysis of osteophyte structure and histology is lacking. Another limiting factor was also difficulty to breed and raise *Postn* homozygous mice due to post-natal death of mice. Due to limiting number of mice available “sham” procedure could not be performed and could be a superior control for this model. Lastly, as we performed bulk RNA-seq only on primary chondrocytes, the findings should be interpreted within the context of cartilage. Since OA is considered a whole-joint disease and Postn plays a role in the bone and probably other parts of the joint, the lack of data from other tissue and cell types is a limitation.

## Conclusions

In summary, *Postn* genetic loss-of-function protects against DMM-induced post-traumatic and age-related spontaneous OA. Our data identify Postn as a novel therapeutic target to delay or prevent OA, independent of the cause despite aforementioned limitations. Further mechanistic studies are warranted to investigate the tissue-specific role(s) of Postn in OA and its impact on other knee tissues such as meniscus and ligaments. Postn modulating therapies will have to be identified and tested before these findings have translational value in the clinical setting.

## Supplementary Information

**Additional file 1.** Gene transcripts

## Data Availability

RNA-seq data have been deposited to the GEO and are accessible through the accession number GSE164534 at https://www.ncbi.nlm.nih.gov/geo/. The other datasets generated and/or analyzed during the current study are not publicly available due to the limit of storage space but are available from the corresponding authors on a reasonable request.

## Abbreviations

Postn: Periostin
OA: Osteoarthritis
wt: Wild type
DMM: Destabilization of the medial meniscus
MMP-13: Matrix metalloproteinase 13 (collagenase 3)
PCR: Polymerase chain reaction
ELISA: Enzyme-linked immunosorbent assay
OARSI: Osteoarthritis Research Society International
μCT: Micro-computed tomography
PBS: Phosphate-buffered saline
NGS: Normal goat serum
COL-2: Collagen type II
DAPI: 4′, 6-Diamidino-2-phenylindole
BV/TV: Bone volume fraction
vBMD: Volumetric bone mineral density
Tb.Th: Trabecular thickness
Tb.N: Trabecular number
Tb.Sp: Trabecular spacing
FBS: Fetal bovine serum
DMEM: Dulbecco’s modified Eagle’s medium
RIN: RNA integrity number
FDR: False-discovery rate
GO: Gene ontology
CI: Confidence interval
ECM: Extracellular matrix
*Evx2*: Even skipped home box 2
*Dscaml1*: DS cell adhesion molecule like one
*Gdf10*: Growth differentiation factor 10
*Tm4sf1*: Transmembrane four superfamily member 1
*Ndufs5*: NADH:ubiquinone oxidoreductase core subunit S5
*Srsf10*: Serine/arginine-rich splicing factor 10
*Gapdh*: Glyceraldehyde-3-phosphate dehydrogenase
ADAMTS: A disintegrin and metalloproteinase with thrombospondin motif
NF-κB: Nuclear factor kappa-light-chain-enhancer of activated B cells
DDR-1: Discoidin domain receptor 1
IL-1β: Interleukin 1 beta
Sox-9: SRY (sex-determining region Y) box transcription factor 9
DAB: 3.3'-Diaminobenzidine
